# Including audience response systems in debriefing. A mixed study during nursing simulation-based learning

**DOI:** 10.1186/s12912-023-01499-z

**Published:** 2023-10-03

**Authors:** Alonso Molina-Rodríguez, María Suárez-Cortés, César Leal-Costa, María Ruzafa-Martínez, José Luis Díaz-Agea, Antonio Jesús Ramos-Morcillo, Ismael Jiménez-Ruiz

**Affiliations:** Department of Nursing, Faculty of Nursing, University of Murcia, Murcia, Spain

**Keywords:** Nursing students, High fidelity simulation training, Gamification, Qualitative research, Quantitative researc

## Abstract

**Background:**

The audience response systems are being implemented to support active learning in nursing degree programs. The benefits of audience response systems have been studied in lecture-based classes and seminars, but their advantages or inconveniences when included in the debriefing phase of a high-fidelity clinical simulation have not been explored. The study aim was to discover student´s experience about using of interactive questions during debriefing, and the self-perceived effects on attention, participation and motivation.

**Methods:**

A Mixed-methods study was used exploratory sequential design in a university. The participants were 4th-year students enrolled in the Nursing Degree in a university in Southern Spain. (1) Qualitative phase: a phenomenological approach was utilized, and focus groups were used for data-collection. (2) Quantitative phase: cross-sectional descriptive study using a questionnaire designed “ad hoc”, on the experiences on the use of interactive questions in the debriefing phase and the Debriefing Experience Scale.

**Results:**

(1) Qualitative phase: the students highlighted the facilitating role of the interactive questions during the reflection part of the debriefing, and mentioned that the interactive questions helped with stimulating attention, participation, and motivation during the analytical part of the debriefing; (2) Quantitative phase: it was observed that the best evaluated dimension was “Motivation”, with a mean of 4.7 (SD = 0.480), followed by the dimension “Participation”, with a mean of 4.66 (SD = 0.461), and lastly, the dimension “Attention”, with a mean of 4.64 (SD = 0.418).

**Conclusions:**

The use of interactive questions contributed the attention, participation, and motivation of the students during the debriefing, contributing towards a highly satisfactory experience of high-fidelity clinical simulation.

**Supplementary Information:**

The online version contains supplementary material available at 10.1186/s12912-023-01499-z.

## Introduction

Clinical simulation as a teaching method for nursing degree students has been used since the start of the 20th Century [1]. However, the medium and high-fidelity simulations were not implemented in nursing studies until the end of the 1990s, with their implementation accelerating in the early 2000’s [2]. In the last few years, high-fidelity clinical simulations have started to be used in Nursing, providing benefits such as the increase in the satisfaction of the students [3], and the improvement in communication skills [4]. The methodology of high-fidelity clinical simulation is composed by four phases (pre-briefing, briefing, simulation scenario, and debriefing), guided by a facilitator [5] with experience in simulation, to guide, to provide support, and to structure the discussion on the learning objectives, and to create a safe environment [6]. In the debriefing phase, the facilitator stimulates the self-reflection and critical thinking skills of the students [7]. Debriefing has been framed within the experience-based learning theory from Kolb, which defines a cyclical learning process in which students acquire knowledge through experience and reflective observation [8]. This process of reflection is a key piece in this theory [9], and promotes the transfer of knowledge, skills, and attitudes to improve safety and the quality of care [5].

Many debriefing techniques and models exist, although the evidence does not point to the superiority of one over the other [10]. One of the most utilized methods, which also works well with the Kolb theoretical model, is the “gather, analyze, and summarize” (GAS) model, which consists of debriefing structured in three parts. These parts explore how the simulation developed, the behavior related with the education objectives is analyzed critically and reflectively, and lastly, the most important aspects of the scenario are summarized [10]. All the techniques and models available share common elements to guarantee a safe environment for the students and to establish quality standards, based on the recommendations from the International Nursing Association for Clinical Simulation and Learning (INACSL) [5]. These elements include establishing rules during the debriefing, such as confidentiality, that encourages students to participate, and the use of open-ended questions. These types of questions help facilitate self-reflection and stimulate the participation of students [11]. Furthermore, during the debriefing, it is important to create an environment that encourages student participation and fosters motivation to learn, while maintaining their attention throughout the entire debriefing phase [12].

In the last few years, the use of new technologies has allowed the implementation of electronic resources, virtual classes, applications with multimedia elements, etc. Multimedia elements have provided advantages through the gamification of the classroom and active learning, as shown by a recent systematic review that described how gamification improved the acquisition of knowledge and the attitude towards learning [13]. To stimulate the participation of students in the classroom, different audience response systems (ARS) have emerged, such Wooclap or Poll Everywhere, which are based on active learning [14]. These systems allow students to ask questions during the class and immediately analyze the results, making the students feel more involved [15]. Access to ARS is through smart phones, tablets, or computers through the internet, which ease its use without requiring additional hardware, and improve the understanding and critical thinking of the students [14].

The development of multimedia elements has led to the emergence of other methods of debriefing in high-fidelity clinical simulation, such as synchronous virtual debriefing, or self-debriefing, which utilizes chats on the internet, blogs, or discussion forums [16, 17]. In synchronous virtual debriefing, questions are included after the simulation through videoconference platforms in small groups of students, while self-debriefing requires the student to individually answer questions after the simulation, allowing for reflection and critical thinking. Both methods have been shown to be valid and efficient for these new debriefing modalities, despite the lack of a facilitator [18, 19].

Until now, multimedia elements have been used during virtual debriefing, but they have not been implemented as a classroom method to contribute to debriefing. The use of interactive questions as a teaching support during debriefing has not been explored in previous studies either. However, their use could provide the advantages offered by the gamification of the content in the high-fidelity clinical simulation debriefing through the ARS in the classroom. To provide new evidence, a study was designed whose objectives were: (1) To discover the experience of students with respect to the use of the Wooclap platform of interactive questions during debriefing, and (2) To quantify the impact of the interactive question on students’ attention, participation, and motivation during debriefing.

## Method

### Design

Mixed-methods study with an exploratory sequential design, in which the qualitative and quantitative phases were conducted in parallel and the conversion of the results from both phases was performed during the interpretation of the results [20, 21].

For the qualitative phase, a phenomenological approach was used to try to capture both individual and collective lived experiences [22], during the high-fidelity clinical simulation sessions. The phenomenological approach is dominated by conversational data collection techniques such as interviews [22, 23]. In this study, focus groups were chosen as a data collection tool to capture the collective experience of the students. These focus groups were used to explore the experiences of the nursing students and included interactive questions in the debriefing phase of the high-fidelity clinical simulation. The consolidated criteria for reporting qualitative research (COREQ) guidelines were used to present our study [24]. For the quantitative phase, a cross-sectional descriptive study was used with a questionnaire designed “ad hoc” (Annex II), on the experiences with the use of interactive questions in the debriefing phase of a high-fidelity clinical simulation, and the Debriefing Experience Scale (DES) [25].

### Setting and participants

The participants were 4th-year students enrolled in the Nursing Degree at the Faculty of Nursing at the University of Murcia, who took the Practicum 4 class during academic year 2021/2022.

The following inclusion criteria were defined as: (1) Having attended the 4 high-fidelity clinical simulation sessions that were part of the class, and (2) Accepting to participate in the study. The exclusion criteria were students enrolled in the European Region Action Scheme for the Mobility of University Students (ERASMUS) program, and students enrolled in national mobility programs (SICUE/SENECA) who would take the class at another university.

To select the participants, students who met the inclusion and exclusion criteria were invited during the months of February and March to participate in the focus groups. The students who wanted to participate were included in a group until data saturation was reached. Student participation was voluntary, and they did not receive any compensation for it. As for the sample size, it was determined as a function of information power [26], and therefore, the eligibility criteria were established to closely associate the characteristics of the sample with the objective of the study. The total number of students enrolled in the class was 223 students, of which 212 met the inclusion and exclusion criteria. Following these criteria, 29 students participated in the qualitative phase of the study, in 4 groups. with the number of participants oscillating between 7 and 8.

The quantitative phase used the same participation criteria as the qualitative phase, although only 160 students, from the initial 212, who met the inclusion and exclusion criteria of the entire class, correctly completed the instruments, for a response rate of 75.4%.

### Intervention

During their 4th year in the Nursing Degree, the students must take the compulsory class Practicum 4, which deals with services such as delivery room, neonatal intensive care unit, hemodialysis, and mental health centers. It is worth 12 European Credit Transfer and Accumulation System (ECTS) credits, and 300 h of work for the students, of which 10 h correspond to high-fidelity clinical simulation and 240 h of real clinical practice. Those students had received 10 h of high-fidelity simulation in a previous year of the program. The simulation laboratories were structured into 4 sessions composed of 18–20 students. In the first session, the scenarios and the principles of simulation were presented, with the structured simulation of 2 scenarios each taking place in the rest of the sessions. The content of the scenarios were mother-child health, mental health, and hemodialysis (Annex I). For the creation of the scenarios, the standards from the (INACSL) were followed [5].

The Wooclap tool was utilized during the debriefing (Wooclap SA, Brussels, Belgium) [27], which objective is to gamify the debriefing phase. This a participative tool, framed within the set of tools known as ARS. It is accessed through a web interface or text messages with mobile devices or computers. The facilitator created different activities in Wooclap, such as multiple-response questions, word clouds, polls, answers through images, etc., with contents specific to the scenarios, which were later used during the debriefing as supporting material.

### Data collection and instrument

In the qualitative phase, the data collection process took place through focus groups guided by a sole interviewer who was the class’ professor and had previous experience in managing focus groups. For the development of the focus group, a guide with a list of themes was created (Table 1). Each focus group lasted between an hour and fifteen minutes, to an hour and a half. The interviews were recorded in audio and video by two cameras. Field notes were collected during and after the focus groups. The focus groups took place between the months of April and May 2021. During the focus groups, the interviewer was only limited to introducing the themes and asking open-ended questions. The interviewer was backed by an observer, who helped to invigorate the groups by asking the participants about their responses. This allowed them to critically reflect on their experiences in retrospect, with a deep interpretation of their experience in the high-fidelity clinical simulation. During the sessions, the researcher performed a reflective process with the participants, in which through paraphrasing and summaries, the students were asked for clarifications to guarantee the understanding of the narrative. The focus group sessions began with a welcome activity, after which they were asked to open the debate, with all the participants able to express their opinions and thoughts about the theme, highlighting the importance of the participation of each of them in the discussion. Afterwards, the norms were established, underlining the non-talking when another person was talking, and lastly, they were informed that the debate could take place through orienting questions about the use of interactive questions in the debriefing phase of the high-fidelity clinical simulation.

**Table 1:** Questions asked in the interviews of the focus groups

How do you think the interactive questions during the debriefing influenced your attention?
How do you think the interactive questions influenced participation during the debriefing?
As for the interactive questions, do you think they can influence motivation in the debriefing?
How can the interactive question influence learning and the assimilation of the concept?
Does having the mobile phone during the debriefing to answer interactive questions have an influence?
What do you think about using the interactive questions to support the completion of the plus/delta?
Can open-ended or close-ended questions influence participation?
With respect to the types of interactive questions, what do you think about the types available?
As for the competition-based or non-competition-based questions, how can these influence participation?
If it had been with other programs, or if the competition mode had been activated, how do you think this could have influenced participation?

Quantitative data collection was carried out by means of an online questionnaire. To complete the questionnaire and the DES, a single form was created in the Google Forms platform, including both instruments. This form was distributed through the internet platform Aula Virtual from the University of Murcia, to the students who met the inclusion and exclusion criteria of the study. The data collection took place between May 15th and June 5th, 2022. In the quantitative phase, in addition to the variables gender and age, the following data collection instruments were used:

- To assess the use of interactive questions in the debriefing phase, an “ad hoc” questionnaire was created. It was based on the results obtained from the focus groups, specifically focusing on topic 2, “Interactive questions in the dynamics of the debriefing.“ with 3 dimensions and a total of 12 items with a Likert scale with 5 response options (where 1 indicates complete disagreement, and 5 complete agreement). Dimension 1 “Attention” measures the attention of the students during the debriefing, and was composed of items A1, A2, A3, and A4. Dimension 2 “Participation” measures the participation of the students, and was composed by items P1, P2, P3, and P4, and dimension 3 “Motivation”, measured the student’s motivation, and was composed by items M1, M2, M3, and M4. Each of the dimensions had a maximum score of 20 and a minimum of 4. All the items were written in the positive sense, so that a higher score indicated a better evaluation of the variable studied. The questionnaire was created and designed by a panel of 3 experts with experience in high-fidelity clinical simulation. To obtain the content validity, 10 educators with more than 2 years of experience in high-fidelity clinical simulation were used, with a resulting content validity index (CVI) being adequate for all the items (CVI between 0.8 and 1), as well as for the total questionnaire (CVI = 0.975). As for the reliability analysis, the internal consistency of the scale was analyzed (Cronbach’s α), which provided a value of α = 0.616 for dimension 1, α = 0.621 for dimension 2, α = 0.796 for dimension 3, and α = 0.875 for the total scale [28].
- Debriefing Experience Scale. To evaluate the experience during the high-fidelity clinical simulation, we used the DES developed by Reed [25], and validated to the Spanish context by Farrés-Tarafa [29]. The instrument was specifically created to evaluate the experience during debriefing, and the importance of high-fidelity clinical simulation. It is composed of 4 dimensions and a total of 20 items that are scored with a Likert scale of 5 points (where 1 indicates complete disagreement, and 5 complete agreement) [29]. Dimension 1 “Learning and making connections”, was composed by items D1.1 to D1.8, dimension 2 “Analyze thoughts and feelings”, was composed by items D2.9 to D2.12, dimension 3, “Facilitator skills in conducting the debriefing” was composed by items D3.13 to D3.17, and lastly, dimension 4, “Appropriate facilitator guidance”, was composed by items D4.18 to D4.20. As for the analysis of reliability, the internal consistency (Cronbach’s α) of the scale was analyzed, based on the scores of the participants in the questionnaire. The following scores were found: dimension 1: α = 0.909, dimension 2, α = 0.778, dimension 3, α = 0.792, dimension 4, α = 0.884, and α = 0.945 for the total scale.

### Data analysis

The focus groups were transcribed verbatim, and afterwards, a preliminary analysis was conducted, composed of a summary of the findings, the interpretations, and the observations with respect to the dynamics of the meeting. This preliminary summary served to afterwards proceed with the thematic analysis following the inductive-deductive procedure proposed by Fereday and Muir-Cochrane [30], for the coding, categorization, sub-categorization, and establishment of a relationship between categories. The process of identifying themes was based on the preliminary codes identified in the literature and in the reading and rereading processes. From here, codes were incorporated inductively, modifying and creating new categories until the configuration presented in the results was reached. To analyze the information, a database was created using MAXQDA software version 12 for Windows. During the research we addressed several issues to increase methodological rigor: we highlight the combination of several data collection methods, data collection until theoretical saturation is reached, and triangulation of researchers. As for the comparison between researchers, all the steps of the analysis process were performed by two researchers, who conducted each phase separately, seeking agreement after each one. Thus, two thematic categories emerged from the data, which backed the use of interactive questions in the high-fidelity clinical simulation.

To process the quantitative information, a database was created in the statistical package JAMOVI version 2.3.9 for Windows. The descriptive statistical values (mean and standard deviation) of the quantitative variables were calculated, as well as frequencies and percentages for each of the answers provided in each of the items of the questionnaire.

### Ethical considerations

The study was approved by the Ethics Committee from the University of Murcia (3640/2021). Informed consent was approved by ethics committee and obtained from all the participants. All the procedures were performed in agreement with the ethical guidelines from the Declaration of Helsinki [31]. The participation of the students was voluntary, after the aim of the study and the ethical guarantees were explained and informed consent was obtained from all the participants. The anonymity of the participants was maintained, and the confidentiality of the data obtained was ensured through the creation of a personal code.

In addition, at the start of the focus groups, information was provided about the ethical aspects and the commitment with confidentiality and privacy. A pact was made to provide each participant with a personal code, and a verbal commitment was agreed upon by the participants.

## Results

The sociodemographic characteristics of the sample show that the average age of the participants was similar in both the quantitative and qualitative phases. The majority of the subjects were women (Table 2).

**Table 2:** Sociodemographic characteristics in both phases

		Quantitative phase	Qualitative phase
Age (Mean; SD)		23.37 (6.22)	22.86 (3.37)
Sex %(N)	Female	84.9% (136)	85.2% (23)
	Men	15.1% (24)	14.8% (6)

### Qualitative phase

Two main themes emerged from the analysis of the data: (1) Interactive questions as support during the debriefing, and (2) Interactive questions in the dynamics of the debriefing. These themes contributed towards the understanding of the experience associated with the use of interactive questions during the debriefing phase of the high-fidelity clinical simulation.

1. Interactive questions as support during the debriefing.

The interviewees highlighted the facilitating function of the interactive questions during the reflective part of the debriefing. The interactive questions helped in the mental reconstruction of the important events during the simulation of the scenario, stimulating their analysis through its recreation through a mental image.

“Also, right when you are here in the debriefing, the questions make you focus more on what you have done, because sometimes you don’t remember if you only start to talk in the debriefing to see what happened.”(S1,29March).

“Sometimes you don’t remember what happened in the scenario, so the questions help you remember what happened and what you did in the simulation room.”(S6,29March).

Likewise, the interactive questions helped as support for the plus/delta resource, as they directed the analysis towards more specific aspects, and helped in the more precise analysis of events in the clinical simulation.

“In the debriefing, they can tell you a thousand things, that you may not remember, but the questions make you aware about what happened in the scenario, and you see what you did well, and you could improve.”(S1,29March).

The analysis of the scenarios through interactive questions as the guiding thread in the debriefing served for the assimilation of learning objectives defined for each scenario through a process of self-reflection focused on the important events of the scenario.

“[…] we learn with the questions, because you ask them as the debriefing moves forward, and we truly focus on the most important aspects and concepts, […], it’s like we focus on the specifics, and we do not leave anything important out. And there are always things that we should remember, and I think the questions work very well for this […].”(S3,5April).

“Also, when the analysis is being done in the debriefing, asking the questions makes us focus on the most important aspects of the scenario, and we remember these in the end, reflecting about the most important things instead of the entire scenario […].”(S3,5April).

As for the type of interactive question utilized, the participants affirmed that the combined use of various formats favored the reflective process during the analysis of the events. The free response questions, and prioritizing the interventions, allowed for more time for self-reflection.

“For me, the ones about prioritizing are harder […] but you learn more, because when you have to take part in the scenario, or you are working […], you know what comes first and what is the most important. I also like the free response ones, because, for example, you can review what you know without looking at the possible options, and so you think more.”(S2,5April)

“[…] for example, the ones about prioritizing […] they help a lot, because on many occasions we have so much knowledge that you have to classify and prioritize them according to the scenario and the actions, and these questions make you reflect about it.” (S5,5April).

“I think that the multiple-choice ones give you more security, because it’s either one or the other, but the free response ones, as she said, give you more possibilities, and allow you to see what the other colleagues wrote down. And the prioritizing ones, I personally think that they make you learn the most.”(S6,5April).

On the other hand, the interactive questions allowed the students to see the other student’s answers at the same time on a screen, thereby enriching the process of the reflective phase of the debriefing, as other options were explored.

“With the question, you can see what could happen according to what the studies say, because comments are made on each of the answers that are shown.”(S4,5April).

2) Interactive questions in the dynamics of the debriefing.

The interviewees indicated that the interactive question helped to stimulate their attention, participation, and motivation, during the analytical phase of the debriefing within a safe environment, while also serving as support for the plus/delta resource.

The use of interactive questions added a series of extrinsic rewards associated to dynamism, competitiveness, or an attractive interface of the tool, to the intrinsic motivation of the students.

*“*What motivates me is that it is interactive […], all of them are answering some questions, and we can see everyone’s opinions, and also we go step by step, it’s a more dynamic way of doing things [subject 2 agrees], so that for me […] they invigorate the entire debriefing when it’s done this way.”(S6,30March).

The fact of knowing how to answer the questions and feel valid and valued by their peers helped with competitiveness in a safe and controlled environment. This promoted the independent work of the students through their prior preparation of the theoretical cross-sectional content of the scenarios.

“It is the first time in four years of the degree that I come prepared to a simulation, in the end, we are all participating, because you know what you have to answer and that there will be a part in which they will ask you questions about the scenario, and you say I’ll take a look and write down the main aspects […].”(S5,5April).

“[…] as for the questions, I think of them as being very motivating, the fact of asking questions and knowing what to answer. Hey, look, I don’t know this. But in the end, I’m not that bad.”(S5,23March).

The interactive questionnaire tools allow for the establishment of classifications during and after completing the questionnaire, increasing the extrinsic motivation of the students through competition between them.

“[…] the top [places] came out, and this motivated me a lot, you want to go out there a do well, and maybe otherwise you say you will answer, and well… […].”(S5,29March).

“[…] if someone comes out, he or she will be in the top places, but the order of the bottom ones does not come up. You see who is moving up, and that can motivate, because with the silly thing of who is winning, you try to answer […].”(S7,29March).

This increase of motivation during the debriefing stimulated attention and participation. Programming the question around the most important events and the learning objectives of the scenario helped to center the focus of attention on the reflection around these objectives.

“[…] we really focused on the most important things, and the most relevant concepts that we had to learn when we finished the scenario, so it really helps, it’s like we focus a lot on the specifics and we don’t leave anything important out.”(S3,5April).

“I think that what it does is to improve attention in the simulation, because on many occasions, once you have finished the simulation, you stop paying attention, and with the questions, I think you are more involved”(S3,5April).

In this sense, the students affirmed that the interactive questions on some specific aspect of the simulation stimulated their participation in the debriefing, so that related themes emerged, within a safe environment promoted by the facilitator.

“[…] although we answer with Wooclap, and although we make mistakes, since we can answer later, […] it makes use talk about the subject, and then […] the professors make us feel comfortable, so that we can talk and not be embarrassed to do it, so that we are comfortable throughout the debriefing.”(S3,5April).

Likewise, the uncertainty about the exact moment in which the question appeared maintained the attention of the students for a longer period. Also, the linearity of the sessions was broken up through a change in type of media utilized (whiteboard, screen, spoken discussion…), and center of attention. Each question could introduce a theme.

“Since we don’t know when they question will be asked, we pay more attention, and we are more attentive during the debriefing, in case they ask through Wooclap, and as they ask about the concepts, you pay more attention […].”(S4,5April).

The students who were not directly involved in the simulation scenario, and who played the role of spectators, also saw their attention promoted, which fomented their integration into the debriefing dynamic. The participants affirmed that the interactive questions avoided the disconnection of the observing students.

“[…] on many occasions, if you are only […] observing, or afterwards only listening during the debriefing, that is, if your group did not do the simulation, it doesn’t mean that it doesn’t interest you that much, but you are not waiting for the corrections as if it was you, but since there are questions, and having to stop and think, makes you become more involved, because in the end you are more attentive, because in the end you answer and see what the others have answered.”(S2,5April).

“With the [interactive] questions, you feel obligated to answer, so that not only those who took part in the scenario get to participate in the debriefing, because on many occasions, only those who took part in the simulation get to participate. This makes you become more involved.” (S5,29March).

The configuration and format of the interactive questions was an important element of analysis in the dynamics and functioning of the debriefing guided by these types of questions. In this sense, the multi-answer question format stimulated participation, as compared to open response questions or “word cloud”. The open response questions assumed minimum knowledge of cross-sectional theoretical knowledge about the scenario, and therefore could have inhibited the response if this knowledge was not present.

“To increase motivation, I supposed that close-ended [questions], because if you have no idea, you are not going to answer anything [in the open-ended questions] […]. With the open-ended question, you see that people answer less, because, for example, if you ask something about a subject that no one knows, you are not going to answer anything […]. If it’s something that you could sense, or more specific things, then you can give more answers.”(S5,29March).

This factor was able to be corrected using anonymous responses, given that they created a safe learning environment and favored the complete participation of the group, independently if the student had a more extroverted or introverted personality, aside from reducing the fear of answering incorrectly.

“I think that perhaps it is embarrassing to speak in public, but when you answer in Wooclap, as it is anonymous, sometimes you answer, and in person, that is, directly, you would not provide an answer in case it is wrong, but as it is anonymous, you do it.”(S7,5April).

Lastly, it is necessary to highlight that the use of mobile devices to answer the questions did not greatly interfere with the students’ attention during the debriefing, as the questions stimulated the increase in student participation.

“And obviously having Wooclap on the phone, in the end you have the phone on the table or with you, and you pay attention to the next question that will come up, in the end you are not going to use it on other things that may result in you losing the thread of the simulation.”(S5,5April).

“I think that the more interesting the techniques you use to maintain our attention, it’s true that we have the phone in our hands, but I don’t feel the need to use for anything else or to send messages, I am waiting to answer.”(S3,29March).

### Quantitative phase

The descriptive analysis of the items showed that all the item and dimensions obtained a score higher than 4.5, except for item A4 “The use of the mobile phone to answer the interactive questions does not decrease my attention during the debriefing”, which obtained the lowest score, with a mean of 4.25 (SD = 0.890). On the contrary, the item with the highest score was A2 “I believe that viewing the most important aspects of the scenario, together with the interactive questions, favor my attention during the debriefing”, with a mean of 4.85 (SD = 0.375). When analyzing the results according to dimensions, it was observed that the best evaluated dimension was dimension 3, “Motivation”, with a mean of 4.7 (SD = 0.480), followed by Dimension 2 “Participation”, with a mean of 4.66 (SD = 0.461), and lastly, dimension 1 “Attention”, with a mean of 4.64 (SD = 0.418). More than 90% of the students polled evaluated all the items with a 4 or 5 (in agreement/completely agree) (Table 3).

**Table 3:** Descriptive statistics for the items and dimensions of the poll

Items	Min	Max.	M (SD)	1	2	3	4	5
Dimension 1. ATTENTION	2.75	5	4.64 (0.418)					
A1. I believe that the use of interactive questions in the analytical phase (plus/delta) during the debriefing favors my attention.	3	5	4.71 (0.542)	0 (0%)	0 (0%)	7 (4.4%)	32 (20%)	121 (75.6%)
A2. I believe that viewing the most important aspects of the scenario, together with the interactive questions, favor my attention during the debriefing.	3	5	4.85 (0.375)	0 (0%)	0 (0%)	1 (0.6%)	22 (13.8%)	137 (85.6%)
A3. Stopping the video to answer the interactive questions makes me focus my attention on specific aspects, and more easily assimilate the more important concepts	3	5	4.73 (0.523)	0 (0%)	0 (0%)	6 (3.8%)	31 (19.4%)	123 (76.9%)
A4. The use of the mobile phone to answer the interactive questions does not decrease my attention during the debriefing.	2	5	4.25 (0.890)	0 (0%)	7 (4.4%)	27 (16.9%)	45 (28.1%)	81 (50.6%)
Dimension 2. PARTICIPATION	2.50	5	4.66 (0.461)					
P1. I believe that the use of interactive questions facilitates the analytical phase (plus/delta) during the debriefing.	2	5	4.72 (0.549)	0 (0%)	1 (0.6%)	5 (3.1%)	31 (19.4%)	123 (76.9%)
P2. The competition-type interactive questions stimulate my participation.	1	5	4.52 (0.861)	2 (1.3%)	5 (3.1%)	12 (7.5%)	30 (18.8%)	111 (69.4%)
P3. I believe that responding to issues with anonymous interactive questions increases my participation.	2	5	4.69 (0.624)	0 (0%)	2 (1.3%)	8 (5%)	27 (16.9%)	123 (76.9%)
P4. Stopping the video to answer the interactive questions increases my participation in the analytical phase (plus/delta) during the debriefing.	1	5	4.69 (0.616)	1 (0.6%)	0 (0%)	7 (4.4%)	32 (20%)	1230 (75%)
Dimension 3. MOTIVATION	2.50	5	4.7 (0.480)					
M1. I believe that reflecting on the most important aspects of the scenario through the use of interactive questions during the debriefing increases my motivation towards learning.	3	5	4.76 (0.469)	0 (0%)	0 (0%)	3 (1.9%)	32 (20%)	125 (78.1%)
M2. The use of competitive interactive questions increases my motivation for correctly answering and learning.	1	5	4.6 (0.754)	2 (2%)	2 (2%)	8 (5%)	34 (21.3%)	114 (71.3%)
M3. I believe that responding to issues with anonymous interactive questions motivates me to reflect.	2	5	4.67 (0.659)	0 (0%)	1 (0.6%)	14 (8.8%)	21 (13.1%)	124 (77.5%)
M4. I believe that the use of interactive questions during the analytical phase (plus/delta) during the debriefing motivates me to participate.	3	5	4.75 (0.514)	0 (0%)	0 (0%)	6 (3.8%)	28 (17.5%)	126 (78.8%)

Min = minimum. Max = maximum. M = mean. SD = standard deviation. 1 = completely disagree. 2 = in disagreement. 3 = indifferent. 4 = in agreement. 5 = completely agree

Lastly, the analysis of the descriptive statistics of the DES questionnaire showed that all the items obtained a mean score higher than 4.5. According to the different dimensions, dimension 1 “Learning and making connections” obtained a mean of 4.73 (SD = 0.425), dimension 2 “Analyze thoughts and feelings” obtained a mean of 4.69 (SD = 0.439), dimension 3 “Facilitator skills in conducting the debriefing”, with a mean of 4.69 (SD = 0.443), and dimension 4 “Appropriate facilitator guidance”, with a mean of 4.76 (SD = 0.437). All of the students polled scored all the items with a 4 or 5 (in agreement /completely agree) (Table 4).

**Table 4:** Descriptive statistics for the items and dimensions of the DES questionnaire

Items	Min	Max.	M (SD)	1	2	3	4	5
DIMENSION 1. Learning and making connections	2.63	5	4.73 (0.425)					
D1.1. Debriefing helped me make connections in my learning	3	5	4.79 (0.450)	0 (0%)	0 (0%)	3 (1.9%)	27 (16.9%)	130 (81.3%)
D1.2. Debriefing was helpful in procession the simulation experience	3	5	4.79 (0.477)	0 (0%)	0 (0%)	5 (3.1%)	23 (14.4%)	132 (82.5%)
D1.3. Debriefing provided me with a learning opportunity	3	5	4.79 (0.468)	0 (0%)	0 (0%)	4 (2.5%)	26 (16.3%)	130 (81.3%)
D1.4. Debriefing helped me find meaning in the simulation	1	5	4.62 (0.681)	1 (0.6%)	0 (0%)	12 (7.5%)	33 (20.6%)	114 (71.3%)
D1.5. My questions from the simulation were answered by debriefing	2	5	4.66 (0.613)	0 (0%)	1 (0.6%)	9 (5.6%)	33 (20.6%)	117 (73.1%)
D1.6. I became more aware of myself during the debriefing session	2	5	4.63 (0.610)	0 (0%)	1 (0.6%)	8 (5%)	40 (25%)	111 (69.4%)
D1.7. Debriefing helped me clarify problems	2	5	4.78 (0.497)	0 (0%)	1 (0.6%)	3 (1.9%)	26 (16.3%)	130 (81.3%)
D1.8. The debriefing helped me makes connections between theory and real-life situations	2	5	4.76 (0.511)	0 (0%)	1 (0.6%)	3 (1.9%)	30 (18.8%)	126 (78.8%)
DIMENSION 2. Analyze thoughts and feelings	2.50	5	4.69 (0.439)					
D2.9. The debriefing helped me analyze my thoughts	2	5	4.64 (0.609)	0 (0%)	1 (0.6%)	8 (5%)	39 (24.4%)	112 (70%)
D2.10. The facilitator reinforced aspects of the team’s behavior	3	5	4.67 (0.578)	0 (0%)	0 (0%)	9 (5.6%)	34 (21.3%)	117 (73.1%)
D2.11. The debriefing environment was psychologically safe	2	5	4.76 (0.523)	0 (0%)	1 (0.6%)	4 (2.5%)	28 (17.5%)	127 (79.4%)
D2.12. Unsettled feelings from the simulation were resolved during the debriefing	2	5	4.69 (0.552)	0 (0%)	1 (0.6%)	4 (2.5%)	39 (24.4%)	116 (72.5%)
DIMENSION 3. Facilitator skills in conducting the debriefing	2.60	5	4.69 (0.443)					
D3.13. The facilitator allowed me enough time to verbalize my feelings before commenting	2	5	4.66 (0.613)	0 (0%)	1 (0.6%)	9 (5.6%)	33 (20.6%)	117 (73.1%)
D3.14. The debriefing session facilitator talked the right amount during the debriefing	2	5	4.76 (0.523)	0 (0%)	1 (0.6%)	4 (2.5%)	28 (17.5%)	127 (79.4%)
D3.15. Debriefing provided a means for me to reflect on my actions during the simulation	3	5	4.72 (0.513)	0 (0%)	0 (0%)	5 (3.2%)	34 (21.3%)	121 (75.6%)
D3.16. I have enough time to debrief thoroughly	2	5	4.54 (0.681)	0 (0%)	1 (0.6%)	14 (8.8%)	42 (26.3%)	103 (64.4%)
D3.17. The debriefing session facilitator was an expert in the content area	3	5	4.78 (0.471)	0 (0%)	0 (0%)	4 (2.5%)	27 (16.9%)	129 (80.6%)
DIMENSION 4. Appropriate facilitator guidance	3	5	4.76 (0.437)					
D4.18. The facilitator taught the right amount during the debriefing session	3	5	4.72 (0.528)	0 (0%)	0 (0%)	6 (3.8%)	33 (20.6%)	121 (75.6%)
D4.19. The facilitator provided constructive evaluation of the simulation during the debriefing	3	5	4.75 (0.462)	0 (0%)	0 (0%)	2 (1.3%)	36 (22.5%)	122 (76.3%)
D4.20. The facilitator provided adequate guidance during the debriefing	3	5	4.82 (0.461)	0 (0%)	0 (0%)	5 (3.1%)	19 (11.9%)	136 (85%)

Min = minimum. Max = maximum. M = mean. SD = standard deviation. 1 = completely disagree. 2 = in disagreement. 3 = indifferent. 4 = in agreement. 5 = completely agree

## Discussion

The results from our study provide evidence on the good acceptance of the use of ARS in the classroom, in agreement with similar studies [32, 33]. Likewise, they show that the use of interactive questions promotes attention, participation, and motivation, with similar results obtained in other studies that explore the use of ARS, which highlight that these increased the student’s attention [33], contributed towards increasing participation [34, 35], and the understanding of the knowledge [35–37]. Multimedia elements have provided advantages through the gamification of the classroom and active learning, as shown by a recent systematic review that described how gamification improved the acquisition of knowledge and the attitude towards learning [13].

Contributions towards the increase in attention, participation, and motivation of the students during debriefing, using interactive questions, is highly important in university education, as the debriefing phase is the cornerstone of high-fidelity clinical simulation, in which the students consolidate the knowledge and competences they just learned.

As for dimension 1 *Attention*, the results show that 95.6% of the students considered that the use of interactive questions favored their attention during the debriefing phase. In the focus groups, the students related the increase in attention with the use of interactive questions. The interactive questions maintained the observing students connected to both the development of the scenario, as well as the debriefing, to correctly answer the questions. As for attention in the classroom, studies on ARS indicate that these types of interactive questions, and the use of applications, favor the attention of the students [38, 39]. Likewise, both the qualitative and quantitative results from our study reconfirm that the use of the smartphone as a teaching tool is not a distracting factor during the debriefing, and other studies on this aspect point to the mobile phone as supporting tool in classrooms [37, 40]. The fact that the use of interactive questions increased the attention of the students during the debriefing is important, as it is in this phase of the high-fidelity clinical simulation in which the student, thanks to the debate that takes place in the classroom, reflects and interiorizes the knowledge acquired [9].

As for dimension 2 *Participation*, the results obtained through both methodologies applied coincided in that the use of interactive questions fomented the participation of the students, and that anonymity increased it. Our study points out that the interactive questions increased participation through three mechanisms: increasing the attention of students who did not take part in the simulation scenario; favoring emergent themes; making use of the multi-response and anonymous questions. Our results coincide with those obtained in studies on the participation through ARS in classrooms [41, 42]. The anonymous nature of the questions must be underlined, as the students indicated that their use made them less embarrassed to speak in public, so they participated more. The participation of the students during the debriefing phase is highly important, as it contributes to the generation of debate and helps when delving into the themes of interest [9].

Dimension 3 Motivation obtained the highest score, with a total of 4.7/5. Most of the students believed that the use of interactive questions contributed towards increasing their motivation to learn how to correctly answer, to reflect, and to actively participate in the debriefing, with similar results obtained in studies on the use of interactive questions [41–43]. The explanation for the high motivation could be that the interactive questions acted as a source of extrinsic motivation, due to the interface itself, as well as the establishment of competition as a gamification mechanic during the debriefing, and the instant gratification, which are aspects of the millennial culture [44]. However, one study found that the use of ARS can have a negative influence when there is too little time to answer questions, leading to a lack of reflection on the part of the students [45].

In addition, the results obtained in the DES scale show that using the intervention in our study, a highly satisfactory experience was achieved during debriefing. The score of the DES was higher as compared to previous studies, so that the use of interactive questions could be related to an improvement in the debriefing experience [46].

### Limitations

The limitations of the present work, which could restrict possible conclusions, include the lack of representativeness, due to the convenience sampling method and the cross-sectional study design utilized. Due to the cross-sectional design, a pretest was not employed on the sample before the intervention, which restricts the outcomes. In addition, the study utilized a more observational methodology, as an experimental design was lacking, which does allow for the establishment of a causality relationship between the use of interactive questions with the benefits described.

## Conclusions

Gamification through ARS during the debriefing phase significantly enhances the overall experience of nursing students. The use of interactive questions increases the attention, participation, and motivation of the students during the debriefing, contributing to a highly satisfactory experience during the high-fidelity clinical simulation. It is important to have innovative tools available, which can be adapted to the new needs of students and allow for critical thinking and the optimization of the debriefing phase in clinical simulation.

### Electronic supplementary material

Below is the link to the electronic supplementary material.

Supplementary Material 1

Supplementary Material 2

## Data Availability

The datasets used and analyzed during the current study are available from the corresponding author on reasonable request.

## Abbreviations

SD: Standard Deviation
GAS: Gather, Analyze, and Summarize
INACSL: International Nursing Association for Clinical Simulation and Learning
ARS: Audience Response Systems
ECTS: European Credit Transfer and Accumulation System
COREQ: Consolidated Criteria for Reporting Qualitative Research
ERASMUS: European Region Action Scheme for the Mobility of University Students
SICUE: Exchange System between University Centers in Spain
SENECA: Aid Program of the Ministry of Education, Culture and Sports to Finance Exchanges in Spain
DES: Debriefing Experience Scale
CVI: Content Validity Index
