# Supplementary material for: Including audience response systems in debriefing. A mixed study during nursing simulation-based learning
Source: BMC Nurs. 2023 Oct 3;22:353. doi: 10.1186/s12912-023-01499-z (PMC10548674; doi:10.1186/s12912-023-01499-z)
Supplement: Supplementary file 1 — Supplementary Material 1 [file 12912_2023_1499_MOESM1_ESM.docx]

Annex I. Simulation scenarios

| TITLE | DESCRIPTION | LEARNING OBJECTIVES |
| --- | --- | --- |
| POSTPARTUM HEMMORRHAGE | 100 ml postpartum hemorrhage with hemodynamic repercussions | -Communication in situations of emergency  -To know the specific treatment of an obstetrics hemorrhage |
| OUT-OF-HOSPITAL DELIVERY | Eutocic delivery with a healthy newborn in an out-of-hospital context | -Anamnesis in a context of emergency  -Initial stabilization of the newborn |
| PUERPERAL CARE | Demand for breastfeeding care related with the newborn’s weight gain | -Knowing the goals of weight gain of the newborn  -Health education with active methodology |
| INFANT CHOKING | FBAO (Foreign-body airway obstruction) of a 9-year-old body which results in CPR | -Apply the FBAO maneuvers  -Basic CPR algorithm |
| BRONCHIOLITIS | Admission and monitoring in neonatology of a newborn with a diagnosis of bronchiolitis. | -Management of newborn’s pain  -Apply oxygen therapy |
| HEMODIALYSIS | Stabilization of APE (acute pulmonary edema) at the start of a hemodialysis session. | -Stabilization of dyspnea  -Start of hemodialysis session |
| BORDERLINE PERSONALITY DISORDER. SELF-HARM THOUGHTS | First visit of an adolescent patient with self-harm ideas to a mental health center. | -Assessment according to Gordon patterns  -Know the self-harm risk scales |
