# Supplementary material for: Including audience response systems in debriefing. A mixed study during nursing simulation-based learning
Source: BMC Nurs. 2023 Oct 3;22:353. doi: 10.1186/s12912-023-01499-z (PMC10548674; doi:10.1186/s12912-023-01499-z)
Supplement: Supplementary file 2 — Supplementary Material 2 [file 12912_2023_1499_MOESM2_ESM.docx]

Annex II. Experiences with the use of interactive questions in the debriefing phase of a high-fidelity clinical simulation.

| Dimension 1. ATTENTION |
| --- |
| A1. I believe that the use of interactive questions in the analytical phase (plus/delta) during the debriefing favors my attention. |
| A2. I believe that viewing the most important aspects of the scenario, together with the interactive questions, favor my attention during the debriefing. |
| A3. Stopping the video to answer the interactive questions makes me focus my attention on specific aspects, and more easily assimilate the more important concepts |
| A4. The use of the mobile phone to answer the interactive questions does not decrease my attention during the debriefing. |
| Dimension 2. PARTICIPATION |
| P1. I believe that the use of interactive questions facilitates the analytical phase (plus/delta) during the debriefing. |
| P2. The competition-type interactive questions stimulate my participation. |
| P3. I believe that responding to issues with anonymous interactive questions increases my participation. |
| P4. Stopping the video to answer the interactive questions increases my participation in the analytical phase (plus/delta) during the debriefing. |
| Dimension 3. MOTIVATION |
| M1. I believe that reflecting on the most important aspects of the scenario through the use of interactive questions during the debriefing increases my motivation towards learning. |
| M2. The use of competitive interactive questions increases my motivation for correctly answering and learning. |
| M3. I believe that responding to issues with anonymous interactive questions motivates me to reflect. |
| M4. I believe that the use of interactive questions during the analytical phase (plus/delta) during the debriefing motivates me to participate. |

Note. Questionnaire designed “ad hoc”
